# Determining Origins and Causes of Childhood Obesity via Mendelian Randomization Analysis

**DOI:** 10.1371/journal.pmed.0050065

**Published:** 2008-03-11

**Authors:** Eric L Ding, Frank B Hu

## Abstract

The authors discuss a new study that investigated the developmental overnutrition hypothesis in a large cohort of British pregnant mothers.

Childhood obesity has become a serious public health problem worldwide. The United States Centers for Disease Control and Prevention estimates that one in three children and adolescents are currently overweight/obese [1]. The adverse complications of childhood obesity on physical health and psychosocial development are tremendous [2]. Moreover, pediatric obesity may substantially increase future burden of cardiovascular disease and other morbidities in adulthood [3,4].

## Developmental Overnutrition Hypothesis

The obesity epidemic not only represents a public health crisis in our current generation, but may also have lasting adverse consequences on the health of future generations. The developmental overnutrition hypothesis proposes that within the pregnant womb of an overweight/obese mother, the developing fetus is exposed to higher in utero levels of glucose and free fatty acids. Such exposure may permanently disrupt or dysregulate appetite control and hormones and impair energy metabolism, thereby increasing offspring adiposity and risk of obesity [5]. This theory suggests that fetal metabolic programming by an adverse in utero environment may lead to obesity in adolescence and young adulthood, spawning a potential vicious cycle of transgenerational transmission of the obesity epidemic from parents to offspring, regardless of offspring genetics or childhood environment.

However, until now, testing such a hypothesis has been exceptionally difficult, as parents exert obvious strong genetic and psychosocial influences on offspring health besides the in utero environment during fetal development. Therefore, although overnutrition of the developing fetus in utero may be one potential explanation of the correlation between maternal and offspring adiposity, the correlation may also be due to shared genetic and social factors. However, cutting-edge methods in genetic epidemiology developed in recent years now allow testing of the hypothesis and of the causal nature of other associations in observational research.

**Linked Research Article**

This Perspective discusses the following new study published in *PLoS Medicine*:

Lawlor DA, Timpson NJ, Harbord RM, Leary S, Ness A, et al. (2008) Exploring the developmental overnutrition hypothesis using parental–offspring associations and *FTO* as an instrumental variable. PLoS Med 5(3): e33. doi:10.1371/journal.pmed.0050033

Using parental–offspring associations and the *FTO* gene as an instrumental variable for maternal adiposity, Debbie Lawlor and colleagues found that greater maternal BMI during offspring development does not appear to have a marked effect on offspring fat mass at age 9–11.

## Investigating the Hypothesis

In this issue of *PLoS Medicine*, Debbie Lawlor and colleagues investigated the developmental overnutrition hypothesis in a large cohort of British pregnant mothers, analyzing 4,091 sets of mother–father–offspring trios [5]. In their initial analysis comparing parental body mass index with offspring fat mass, they found that the association between parental body mass index and offspring adiposity at ages nine to 11 was stronger among mother–offspring pairs than among father–offspring pairs. This finding suggests that the maternal influence on offspring adiposity extends beyond genetic contributions alone—providing some support to the developmental overnutrition hypothesis. However, such evidence for this hypothesis is indirect, as the findings could be explained by the influence of other maternally predominant factors such as breast-feeding. Therefore, Lawlor and colleagues undertook a second, more sophisticated analysis using a previously established gene of adult and childhood obesity, the *FTO* gene [6], as an “instrument” of maternal obesity (a marker for the in utero environment) via a Mendelian randomization (MR) analysis.

## Principles of Mendelian Randomization Analysis

In transferring genes from parents to offspring, genetic alleles are assigned through a random process at the time of gamete formation (Mendel's Second Law). The randomly assorted alleles are analogous to randomly allocated treatments in randomized controlled trials. Based on this principle, MR analysis uses genetic variants with known functions as markers of long-term exposure to estimate unconfounded exposure–outcome associations in observational studies [7]. The analysis requires the identification of a genetic variant that robustly predicts the exposure of interest to serve as an instrumental variable for this exposure, also known as the “intermediate phenotype.” Theoretically, such analysis can eliminate all confounding bias because subjects are randomly assigned to different genotypes, thereby enabling one to determine the potential causal relation between exposure and outcome.

In this study, the authors capitalize upon the *FTO* gene and its established relation to adiposity [6] to elucidate whether maternal overweight/obesity, the intermediate surrogate of the in utero nutritional environment (exposure), is causally related to offspring adiposity (outcome). Using the MR analysis, the authors concluded that the data did not support the developmental overnutrition hypothesis. However, the 95% confidence intervals for the MR estimates were wide, and there are several issues to consider when interpreting the data.

## Strengths and Limitations

The study has considerable strength as a prospective study based on a well-established cohort, with anthropomorphic information from a large number of parental–offspring trios. In addition, objective data on adiposity in offspring were available from dual energy x-ray absorptiometry scans. Although the application of MR analysis in the study has certain advantages in estimating causal associations compared to conventional observational analyses, there are potential limitations [8] that deserve discussion.

The phenomenon of linkage disequilibrium (LD), where one allele is actually a correlated marker for another allele on the same chromosome (i.e., the alleles are nonrandomly associated), is one classical concern. However, LD between neighboring genetic variants can still be acceptable, as many variants are considered markers of a haplotype block characterizing a consecutive region of a gene, thereby representing the net functional effect of a set of alleles. The MR assumption is violated only if the chosen gene is in LD with another gene that has independent effects on the outcome [9]. Another recent study in *PLoS Medicine* by the same group indicated that distant genes and genes on different chromosomes are uncorrelated between each other, indicating that genes are mostly free of confounding by other genes [10].

Another assumption of MR analysis requires that any proposed effect of the gene on outcome should be mediated through its intermediate (i.e., the *FTO* gene is valid as an instrument of MR if the maternal *FTO* gene's effects on offspring obesity are mediated through the in utero environment, as marked by maternal adiposity). However, the assumption is violated if the *FTO* gene possesses pleiotropic effects (other biologic effects) on outcome independent of its effects on maternal adiposity. In such instances, the analysis may not yield the true effect of fetal overnutrition, unless one analytically adjusts for and blocks all alternative causal pathways.

Finally, this study indeed represents a novel application of the MR analysis to transgenerational causal inferences. However, results were strongly dependent on whether analyses were adjusted for offspring *FTO* or not, with the association being drastically altered from positive to null after adjusting for offspring *FTO*. Conceptually, it is necessary to adjust for the offspring *FTO* gene to separate the influence of genetic inheritance of the maternal *FTO* gene from the effect of maternal adiposity during pregnancy. However, as the offspring *FTO* is a causal pathway intermediate between the maternal *FTO* and offspring adiposity, the conventional approach of adjustment through stratification may induce bias in estimating the exposure–outcome association [11]. This methodological issue deserves consideration in future studies.

## Conclusion and Future Directions

Overall, with the unique and large prospective cohort of over 4,000 parental–offspring trios and the integration of genetic data via MR analysis for causal inference, this study presents an important contribution to testing the fetal origin of obesity hypothesis. Not only does this provocative study warrant replication in a different population, but future research should also aim to investigate the fetal origin hypothesis using other direct intermediate measures of in utero “overnutrition,” such as gestational levels of maternal hyperglycemia and free fatty acids. Moreover, it would also be highly informative to investigate the hypothesis via MR using other established or soon-to-be discovered genes responsible for obesity, diabetes, and/or other metabolic disorders. Recent advances in genome-wide association studies have made it possible to identify reproducible genes for these conditions. Larger sample sizes and more robust estimates of the genotype–intermediate phenotype and genotype–outcome associations in future studies would also help provide more precise MR estimates for causal inference.

While Lawlor and colleagues' new study does not support the developmental overnutrition hypothesis, the wide confidence intervals do not provide a clear refutation of the hypothesis either. The fetal origin theory still remains supported by a body of plausible biological mechanisms and substantial empirical data. Indeed, an epidemic mechanism whereby obesity may “accelerate through successive generations independent of further genetic or environmental factors” [12] in a propagating vicious cycle would potentially be difficult to control via conventional medical and public health interventions—thus necessitating a high priority for further investigation.

## Abbreviations

LD: linkage disequilibrium
MR: Mendelian randomization
